# Dementia subtype prediction models constructed by penalized regression methods for multiclass classification using serum microRNA expression data

**DOI:** 10.1038/s41598-021-00424-1

**Published:** 2021-10-22

**Authors:** Yuya Asanomi, Daichi Shigemizu, Shintaro Akiyama, Takashi Sakurai, Kouichi Ozaki, Takahiro Ochiya, Shumpei Niida

**Affiliations:** 1 Medical Genome Center, Research Institute, National Center for Geriatrics and Gerontology, 7-430 Morioka-cho, Obu, Aichi 474-8511 Japan; 2 Department of Medical Science Mathematics, Medical Research Institute, Tokyo Medical and Dental University, Tokyo, Japan; 3 RIKEN Center for Integrative Medical Sciences, Yokohama, Japan; 4 Center for Comprehensive Care and Research on Memory Disorders, National Center for Geriatrics and Gerontology, Obu, Aichi Japan; 5 Department of Cognition and Behavior Science, Nagoya University Graduate School of Medicine, Nagoya, Aichi Japan; 6 Division of Molecular and Cellular Medicine, Fundamental Innovative Oncology Core Center, National Cancer Center Research Institute, Tokyo, Japan; 7 Department of Molecular and Cellular Medicine, Institute of Medical Science, Tokyo Medical University, Tokyo, Japan

**Keywords:** Diagnostic markers, Dementia

## Abstract

There are many subtypes of dementia, and identification of diagnostic biomarkers that are minimally-invasive, low-cost, and efficient is desired. Circulating microRNAs (miRNAs) have recently gained attention as easily accessible and non-invasive biomarkers. We conducted a comprehensive miRNA expression analysis of serum samples from 1348 Japanese dementia patients, composed of four subtypes—Alzheimer’s disease (AD), vascular dementia, dementia with Lewy bodies (DLB), and normal pressure hydrocephalus—and 246 control subjects. We used this data to construct dementia subtype prediction models based on penalized regression models with the multiclass classification. We constructed a final prediction model using 46 miRNAs, which classified dementia patients from an independent validation set into four subtypes of dementia. Network analysis of miRNA target genes revealed important hub genes, *SRC* and *CHD3,* associated with the AD pathogenesis. Moreover, *MCU* and *CASP3*, which are known to be associated with DLB pathogenesis, were identified from our DLB-specific target genes. Our study demonstrates the potential of blood-based biomarkers for use in dementia-subtype prediction models. We believe that further investigation using larger sample sizes will contribute to the accurate classification of subtypes of dementia.

## Introduction

Dementia is a syndrome characterized by loss of cognitive function, in particular memory, thinking, and behavioral abilities. The number of people worldwide with dementia among the elderly population is rapidly increasing, and is expected to reach 74.7 million in 2030 and 131.5 million in 2050^1^. The most common subtype of dementia is Alzheimer’s disease (AD), followed by vascular dementia (VaD), and dementia with Lewy bodies (DLB). The diagnoses of these subtypes of dementia are based on clinical criteria for each subtype^2–6^. However, it is difficult to differentiate between dementia subtypes, because many clinical features overlap. For example, patients with normal pressure hydrocephalus (NPH) present with similar symptoms to AD, although NPH is often curable by shunt surgery to remove accumulated cerebrospinal fluid (CSF)^7^. Therefore, the identification of new biomarkers for more efficient diagnosis is urgently required.

Imaging-based techniques, including positron emission tomography scans for detection of amyloid beta (Aβ) deposition or tau tracers, and volumetric magnetic resonance imaging for determination of hippocampal or medial temporal lobe atrophy, are currently used for diagnosis of dementia subtypes; however, these techniques are not suitable for initial screening due to their high cost^8^. In addition, while several CSF biomarkers, such as Aβ_1–42_, total tau (T-tau), and phosphorylated tau 181 (P-tau_181_), are effective for characterizing AD, these are not suitable for initial screening due to their high invasiveness. Therefore, blood-based biomarkers are the most attractive option as candidate biomarkers for practical clinical use due to their minimal invasiveness and cost effectiveness.

MicroRNAs (miRNAs) are small non-coding RNAs of 20–25 nucleotides in length that regulate gene expression by binding to complementary regions of messenger RNAs^9^. Many circulating miRNAs in blood have been identified as promising biomarkers for cancer diagnosis^10–15^ and dementia diagnosis^16–22^. However, the prediction models constructed for dementia using miRNAs are specific to each subtype^16–22^ and the development of a multiclass classification prediction model of dementia subtypes is required.

Here, we conducted a comprehensive serum miRNA expression analysis of 1348 Japanese dementia patients composed of four subtypes (AD, VaD, DLB, and NPH) and 246 control subjects. Using this data, we constructed various dementia subtype prediction models based on penalized regression models with multiclass classification. Our final prediction model classified dementia patients into four subtypes of dementia using 46 miRNAs. Furthermore, network-based meta-analysis of the miRNA target genes revealed several important hub genes associated with the pathogenesis of dementia subtypes. We believe that further investigation using a larger sample size will contribute to accurate classification of dementia subtypes.

## Methods

### Clinical samples

All 1594 serum samples and clinical information, including sex, age, and *APOE* ε*4* genotypes, were obtained from the National Center for Geriatrics and Gerontology (NCGG) Biobank. Of these samples, 1009 were from patients with AD, 89 were from patients with VaD, 166 were from patients with DLB, and 84 were from patients with NPH; 246 samples were from cognitively normal elder controls (CN). The AD patients were diagnosed based on the criteria developed by the National Institute on Aging and the Alzheimer's Association (NIA-AA)^2,3^; the VaD patients were diagnosed using the criteria in the report of the NINDS-AIREN International Workshop^4^; and the DLB and NPH patients were diagnosed based on the criteria in the fourth report of the DLB Consortium^5^ and the guidelines for the management of NPH^6^, respectively. The CN samples had subjective cognitive complaints, but normal cognition on the neuropsychological assessment with a comprehensive neuropsychological test (Mini-Mental State Examination score, > 25). All individuals were 60 years of age or older. This study was approved by the ethics committee of the NCGG. The design and performance of the study were clearly described in a research protocol. Participation was voluntary and all participants completed informed consent in writing before registering to NCGG Biobank. All methods in this study were performed in accordance with the Declaration of Helsinki.

### miRNA-microarray assay

Total RNAs were extracted from 300 µL serum samples by using a 3D-Gene RNA extraction reagent (Toray Industries, Inc., Kanagawa, Japan). Comprehensive miRNA expression analysis was performed using the 3D-Gene system and a 3D-Gene Human miRNA Oligo Chip (Toray Industries, Inc.), which was designed to detect 2565 of the miRNA sequences registered in miRBase (release 21, http://www.mirbase.org/). All microarray data used in this study are publicly available through the Gene Expression Omnibus database accession numbers: GSE120584 and GSE167559 (https://www.ncbi.nlm.nih.gov/geo/). The miRNA expression normalization was described in our previous study^20^.

### Construction and evaluation of the dementia-type risk prediction model

**Figure 1:**
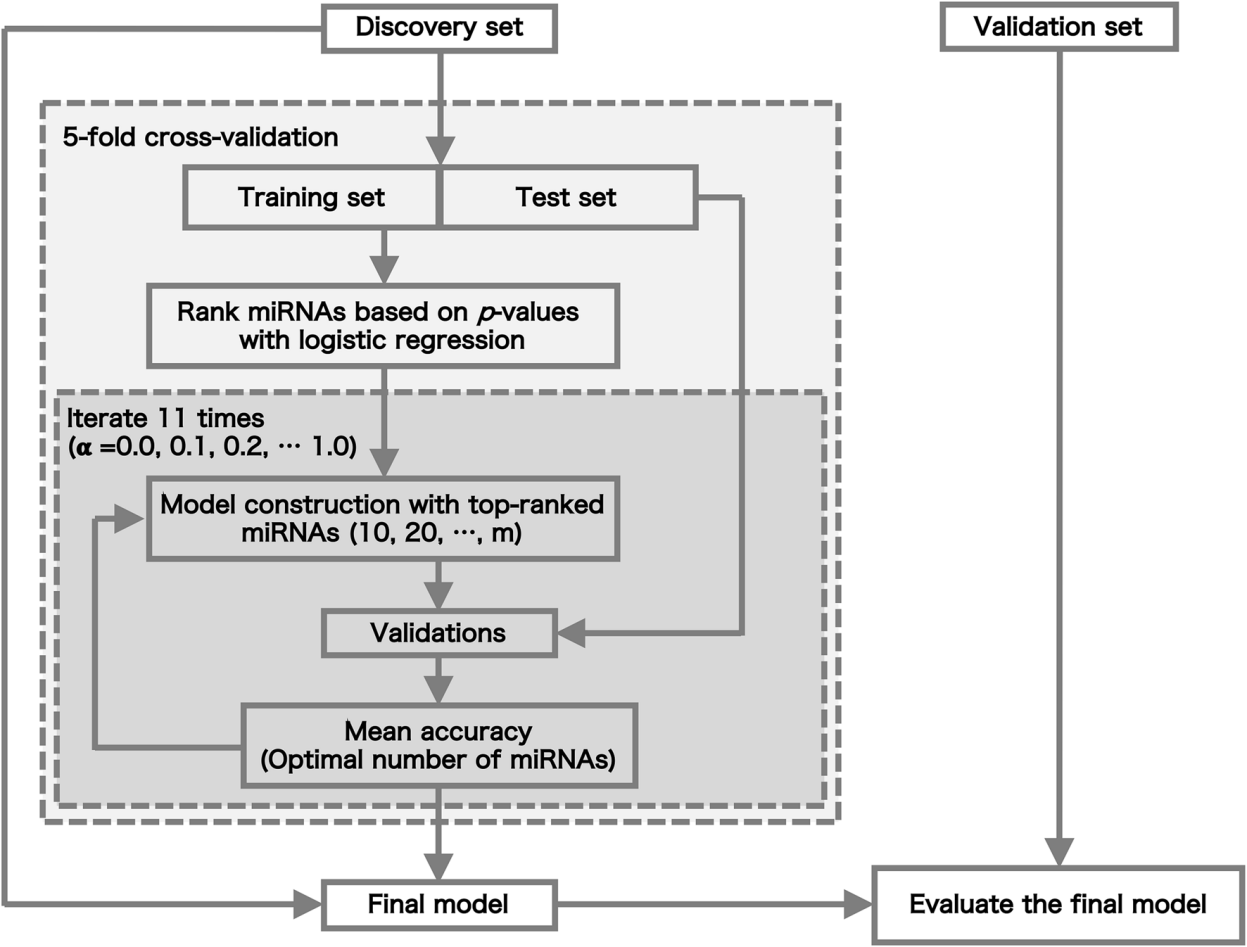
Outline of construction of dementia subtype prediction model and validation.

All data were strictly separated into a discovery set and validation set (Fig. 1). Four-fifths of the discovery set was used to calculate *p*-values in each cross-validation step. The *p*-values corresponding to the miRNAs were calculated with a logistic regression model between dementia and CN with adjustments for three covariates: age, sex, and the number of *APOE* ε*4* alleles. The top-ranked *m* miRNAs were selected stepwise (*m* = 10, 20, …). Using a combination of the *m* miRNAs and three clinical variables, risk prediction models were constructed based on penalized regression methods: ridge regression, elastic net, and lasso. Let $X_{i} = \left( {X_{i,1} , \ldots ,X_{i,p} } \right)$ be the values of the pre-selected top-ranked *m* miRNAs for a subject *i* and let $l\left( {\beta ;\gamma_{i} ,X_{i} } \right)$ be the logistic log-likelihood:

$$l\left( {\beta ;\gamma_{i} ,X_{i} } \right) - \lambda P_{\alpha } \left( \beta \right),$$

where $P_{\alpha } \left( \beta \right) = \left( {1 - \alpha } \right)\frac{1}{2}\beta^{2} + \alpha \left| \beta \right|$ and $\alpha$ is set to 1 (lasso), 0 (ridge regression), or 0.1 to 0.9 at 0.1 intervals (elastic net), and the optimal penalty parameter $\lambda$ is selected using fivefold cross-validation. This process was repeated 5 times (fivefold cross-validation). Based on the average accuracy, we determined the optimal number of miRNAs and $\alpha$ for model construction. The final model was constructed using the entire discovery set and the adjusted model was evaluated on the independent validation set. The multiclass classification model used in this study was implemented using the *glmnet* package (version 2.0–18)^23^ with the function family set to "multinomial" in the statistical software R^24^.

### Network analysis for the target genes of miRNAs

Up- and down-regulated miRNAs were defined based on the coefficients of the multiclass dementia subtype prediction model. The miRNAs with positive coefficients were assigned to up-regulated miRNAs, and those with negative coefficients were assigned to down-regulated miRNAs. The miRNA target gene annotation was conducted using the microRNA Target Prediction and Functional Study Database (miRDB)^25^. Only target genes with a prediction score (assigned by MirTarget V3 based on miRBase 21) of greater than 90 were used in this study. Based on the target genes, protein–protein interaction (PPI) network analyses were implemented using the web-based tool NetworkAnalyst 3.0 (https://www.networkanalyst.ca/)^26^ together with the International Molecular Exchange Consortium Interactome database^27^; we set the betweenness cutoff, which indicates the number of shortest paths through a node, to 25 and used the minimum network filter. The PPI networks were visualized using Cytoscape (version 3.7.1)^28^. We identified nodes with degree > 100 as hub nodes. We identified statistically significant KEGG pathways and biological process gene ontologies (GOs) by using NetworkAnalyst. The *P*-value was corrected for multiple testing using False Discovery Rate (FDR), and the statistical significance threshold was set at FDR < 0.05.

### Ethics approval and consent to participate

This study was approved by the ethics committee of the NCGG. The design and performance of the current study, which involves human subjects, were clearly described in a research protocol. All participation was voluntary, and participants completed informed consent in writing before registering with the NCGG Biobank.

## Results

### Subjects

A total of 1594 Japanese people comprising 1009 AD, 89 VaD, 166 DLB, 84 NPH, and 246 CN subjects, were enrolled in this study in the NCGG Biobank. The male:female ratio of all individuals was 1:1.68 with an average age of 78.0 years (SD, 6.8). The allele frequency of the C allele of rs429358, which defines the *APOE* ε*4* phenotype, was 0.203 (Table 1). We also split the participants into a discovery set and a validation set. This separation was stratified on age and sex such that there was a similar distribution of these features in the discovery and validation sets.

**Table 1:** Sample characteristics of each dementia subtype.

Group	No. of samples	Male:Female	Age (mean ± 1 S.D.)	rs429358^a^			
				TT	TC	CC	MAF^b^
AD	1009	1:2.33	79.3 ± 6.2	565	376	68	0.254
VaD	89	1:0.68	79.1 ± 6.4	67	21	1	0.129
DLB	166	1:1.52	79.5 ± 6.0	115	48	3	0.163
NPH	84	1:0.95	78.9 ± 6.1	69	14	1	0.095
CN	246	1:0.91	71.1 ± 6.2	207	36	3	0.085
Total	1594	1:1.68	78.0 ± 6.8	1023	495	76	0.203

^a^The C allele of rs429358 defines the *APOE* ε*4* phenotype, while the T allele defines wild-type, ε*3.*

^b^*MAF* Minor allele frequency.

### Dementia subtype prediction models

We constructed dementia subtype prediction models based on penalized regression methods for multiclass classification (Fig. 1). We carefully examined the sample size of each dementia subtype in the discovery data set, as the imbalance of sample size in multiclass classification is a well-known issue in the field of machine learning. The detailed procedures of four models, Model A-D, are described in Supplementary Note and Supplementary information (Figs. S1, S2, and Tables S1–S5).

To eliminate the bias of sample size among dementia subtypes, we constructed a final model using the same number of samples in each dementia subtype. We selected 50 samples, composed of 25 males and 25 females, from four dementia subtypes and CN. In total, 250 samples were used for a discovery data set and the remaining samples were used for a validation data set (Supplementary Table S3, Dataset 3). Through the fivefold cross-validation, the maximum mean accuracy for dementia subtypes was achieved with a parameter combination of (*m*, $\alpha$) = (200, 0.9) (Supplementary Fig. S3). The final prediction model, Model D (Supplementary Table S6), was constructed with the optimal parameters detected using the entire discovery set and achieved an accuracy of 0.398 in the validation data set and a mean accuracy of 0.374 for dementia subtypes (Supplementary Table S3) when using 46 miRNAs.

### PPI network analyses using miRNA target genes

**Figure 2:**
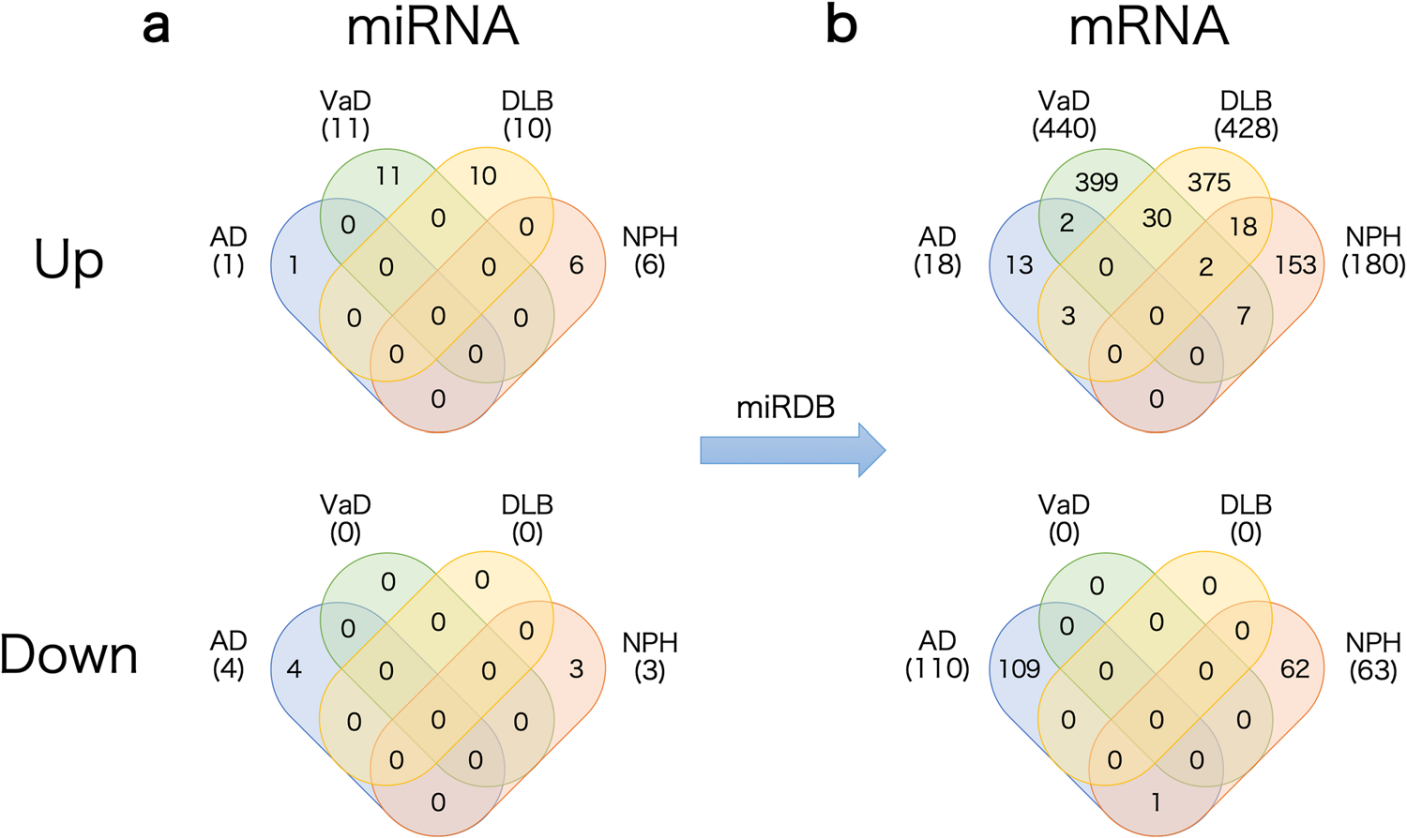
Effective miRNAs and genes used in our dementia-type prediction model. (**a**) The number of up- and down-regulated miRNAs used for the final models for each dementia subtype. (**b**) Target genes for each miRNA used for each model; the target genes were predicted by using annotation in the miRDB data.

**Figure 3:**
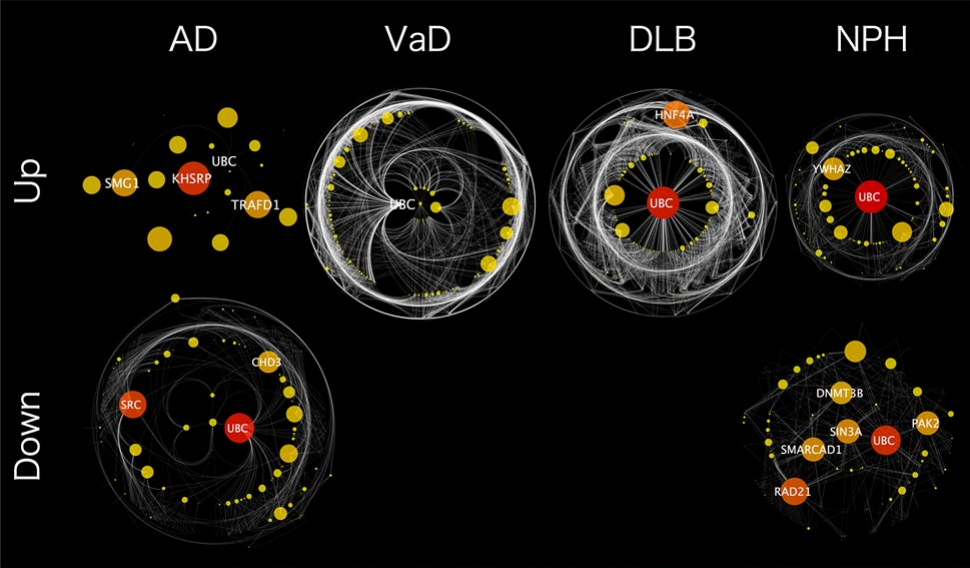
PPI network analysis for the genes that were targeted by the miRNAs. The gene symbol is displayed for nodes with degree > 100.

According to the annotation in miRDB, the 46 miRNAs used in Model D were predicted to target 1303 genes. Of these 1303 genes, the numbers of genes related to up- and down-regulated miRNAs were, respectively, 18 and 110 in AD, 440 and 0 in VaD, 428 and 0 in DLB, and 180 and 63 in NPH (Fig. 2). To elucidate functional modules from the target genes, PPI network analyses were performed using NetworkAnalyst for each dementia subtype; PPI network visualization was conducted with Cytoscape software (Fig. 3). Several interesting hub genes were identified in each dementia subtype. Of them, *SRC* and *CHD3*, which are targets of down-regulated miRNAs in AD, are particularly interesting. The *SRC* gene is reported to be associated with AD^29–31^. The *CHD3* gene is reported to be involved in transcription of the *PSEN1* gene, the most common cause of familial Alzheimer's disease^32–34^. While the *UBC* gene was observed in all dementia subtypes networks, it was not one of the 1303 target genes that we detected.

We further performed gene set enrichment analyses (GSEA) using NetworkAnalyst for each dementia subtype. A statistically significant KEGG pathway (Ras signaling pathway, hsa04014, FDR = 0.0083 in VaD) and four statistically significant GO terms (Nucleobase-containing compound transport, GO:0015931, FDR = 0.029 in AD; RNA export from nucleus, GO:0006405, FDR = 0.046 in AD; Homophilic cell adhesion, GO:0007156, FDR = 5.3 × 10^–5^ in DLB; Cell–cell adhesion, GO:0098609, FDR = 6.6 × 10^–4^ in DLB) were identified in the target genes of up-regulated miRNAs (Table 2).

**Table 2:** Gene set enrichment analyses using the target genes of the miRNAs.

Gene set	Group	Up-/Down -regulated miRNAs	Pathway/GO term	No. of genes (Hits/Total)	*p*-value	FDR^a^
KEGG	VaD	Up	Ras signaling pathway (KEGG: hsa04014)	17/232	2.6 × 10^–5^	0.0083
GO	AD	Up	Nucleobase-containing compound transport (GO:0015931)	4/175	3.5 × 10^–5^	0.029
		Up	RNA export from nucleus (GO:0006405)	3/86	1.1 × 10^–4^	0.046
	DLB	Up	Homophilic cell adhesion (GO:0007156)	17/139	6.5 × 10^–8^	5.3 × 10^–5^
		Up	Cell–cell adhesion (GO:0098609)	30/461	1.6 × 10^–6^	6.6 × 10^–4^

^a^*FDR* false discovery rate.

## Discussion

We report a prediction model for multiclass classification of dementia subtypes using serum miRNAs. Most previous research has focused on developing prediction models for specific dementia subtypes^16–22^. Shigemizu et al*.* reported separate effective blood miRNA-based risk prediction models for several dementia subtypes^20,21^; however, such individual dementia subtype prediction models would likely predict multiple dementia subtypes for each patient. To address this issue, we propose a multiclass classification dementia subtype prediction model. Although our models could not provide sufficient accuracy, a further, larger cohort study is expected to improve the prediction accuracy. In particular, an improvement of the prediction accuracy can be expected in patients with VaD and NPH as the number of affected patients in this study was small.

Network analyses were performed using the target genes of the 46 miRNAs involved in our model that used equal sample sizes for the four subtypes, Model D. Two interesting hub genes, *SRC* and *CHD3*, were detected from the PPI network of the target genes of down-regulated miRNAs in AD. Accumulation of Aβ in the brain is strongly associated with the onset of AD. The constitutively active form of Src increases Aβ generation, and the inhibition of the kinase activity of Src reduces the generation of Aβ in cells stably expressing *APP*^29^. Src also regulates the phosphorylation of Mint, which has an important role in the production and the secretion of Aβ^30,31^. *CHD3* suppresses transcription of the familial AD causative gene *PSEN1*^32,33^ and may potentially rescue Ca^2+^‐signaling defects in familial AD patients and prevent neuronal apoptosis^34^. While *UBC* appeared in all networks, there were no miRNAs targeting *UBC* in our model. This may be because the housekeeping gene *UBC* (Ubiquitin C) has a key role in various pathways, such as DNA repair, cell cycle regulation, kinase modification, endocytosis, and regulation of other cell signaling pathways.

Dementia-subtype–specific target genes could be effective in distinguishing between the four dementia subtypes. Two genes, *MCU* and *CASP3,* that are associated with DLB pathogenesis^35–37^, were identified from the target genes of up-regulated miRNAs in DLB. Calcium uptake via the mitochondrial calcium uniporter (MCU) complex and mitochondrial calcium overload play a key role in neurodegenerative diseases, including DLB^35,36^. Neuronal accumulation of α-synuclein is a major feature of DLB. Desplats et al. reported that neuron-to-neuron transmission of α-synuclein triggers activation of caspase 3, which is coded by *CASP3*, and causes neuronal cell death^37^.

The Ras signaling pathway (hsa04014) was identified through GSEA using target genes of up-regulated miRNAs in VaD. The Ras signaling pathway was also identified from target genes of down-regulated miRNAs in AD, but the result was not statistically significant after FDR correction. Kirouac et al. reported that the expression of Ras is associated with Aβ production in human AD brains^38^. Therefore, further study of genes involved in the Ras signaling pathway could contribute to understanding the difference between AD and VaD. In addition, four statistically significant GO terms (Nucleobase-containing compound transport, RNA export from nucleus, Homophilic cell adhesion, and Cell–cell adhesion) were identified from target genes of up-regulated miRNAs in AD and DLB. The association between these terms and dementia is currently unknown. These GO terms, however, may provide important clues to the underlying mechanisms for AD and DLB pathogenesis.

Blood-based biomarkers, which form the basis of our prediction models are attractive because they are minimally invasive, cost-effective, and easy to reproduce; however, the strategy we present here has a limitation. It is not applicable to mixed dementia cases, in which patients exhibit more than one subtype of dementia simultaneously^39,40^. As long as this issue is unresolved, it may be difficult to apply our findings to practical clinical use.

## Conclusions

We developed dementia subtype prediction models by using penalized regression methods for multiclass classification and serum microRNA expression data. Network analysis of the miRNA target genes revealed several important hub genes associated with AD pathogenesis. Moreover, we found several genes associated with DLB pathogenesis, from our DLB-specific target genes. Our study demonstrates the potential of blood-based biomarkers for dementia subtype prediction models. We believe that further investigation using a larger sample size will contribute to a more accurate classification of dementia subtypes.

## Supplementary Information

Supplementary Figures.

Supplementary Tables.

Supplementary Information.

## Data Availability

All microarray data used in this study are publicly available through the Gene Expression Omnibus database accession number GSE120584 and GSE167559 (https://www.ncbi.nlm.nih.gov/geo/).

## Abbreviations

AD: Alzheimer’s disease
CN: Cognitively normal elder control
CSF: Cerebrospinal fluid
DLB: Dementia with Lewy bodies
GSEA: Gene set enrichment analyses
miRNA: MicroRNA
NPH: Normal pressure hydrocephalus
VaD: Vascular dementia
