# Supplementary material for: Dementia subtype prediction models constructed by penalized regression methods for multiclass classification using serum microRNA expression data
Source: Sci Rep. 2021 Oct 22;11:20947. doi: 10.1038/s41598-021-00424-1 (PMC8536697; doi:10.1038/s41598-021-00424-1)
Supplement: Supplementary file 1 — Supplementary Figures. [file 41598_2021_424_MOESM1_ESM.pdf]

Supplementary Information for

Dementia subtype prediction models constructed by penalized regression methods for multiclass classification using serum microRNA expression data

Yuya Asanomi[1], Daichi Shigemizu[1–3*], Shintaro Akiyama[1], Takashi Sakurai[4,5], Kouichi Ozaki[1,3], Takahiro Ochiya[6,7], and Shumpei Niida[1*]

[1] *Medical Genome Center, National Center for Geriatrics and Gerontology, Obu, Aichi, Japan*
[2] *Department of Medical Science Mathematics, Medical Research Institute, Tokyo Medical and Dental University, Tokyo, Japan*
[3] *RIKEN Center for Integrative Medical Sciences, Yokohama, Japan*
[4] *Center for Comprehensive Care and Research on Memory Disorders, National Center for Geriatrics and Gerontology, Obu, Aichi, Japan*
[5] *Department of Cognition and Behavior Science, Nagoya University Graduate School of Medicine, Nagoya, Aichi, Japan*
[6] *Division of Molecular and Cellular Medicine, Fundamental Innovative Oncology Core Center, National Cancer Center Research Institute, Tokyo, Japan*
[7] Department of Molecular and Cellular Medicine, Institute of Medical Science, Tokyo Medical University, Tokyo, Japan

* Correspondence should be addressed to D.S. (daichi@ncgg.go.jp) or S.N. (sniida@ncgg.go.jp)

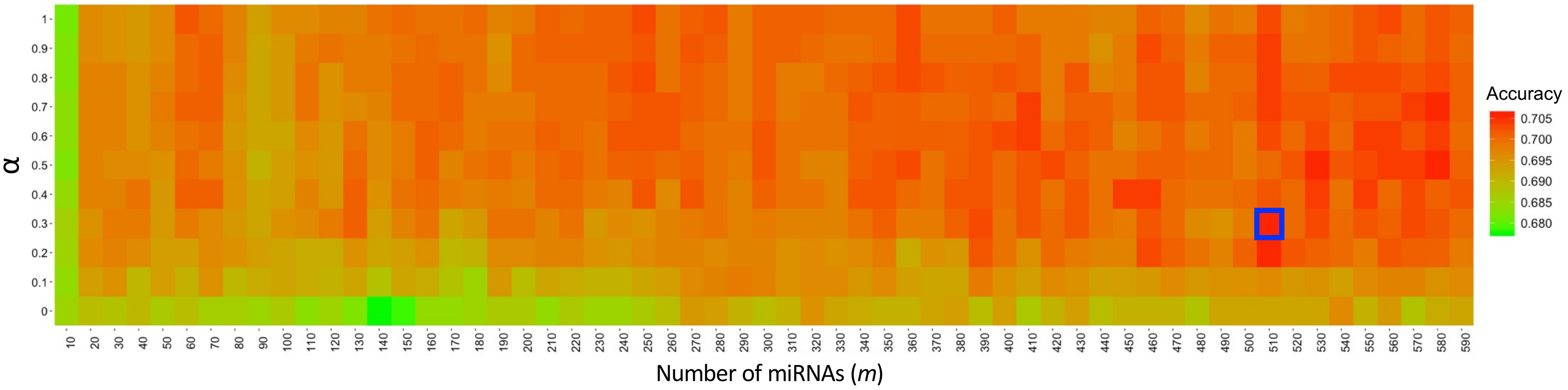

Supplementary Fig. S1. Five-fold cross-validation on a discovery set of Dataset 1.
The x-axis shows the number of miRNAs (m). The y-axis shows α, which is used for penalized regression methods. A combination of $(m, \alpha) = (510, 0.3)$ achieved the highest mean accuracy in the test set (blue rectangle).

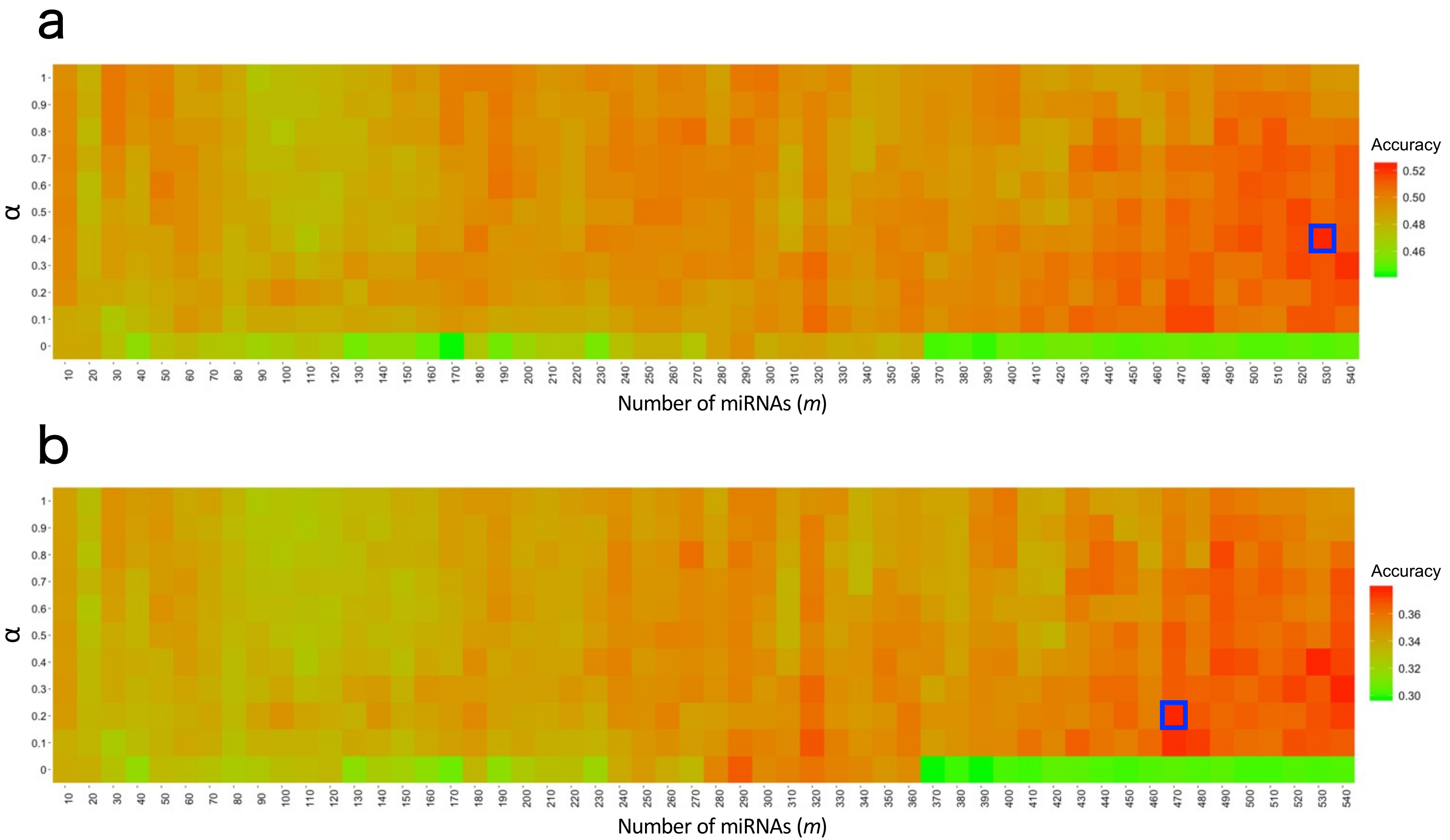

Supplementary Fig. S2. Five-fold cross-validation on a discovery set of Dataset 2.
The x-axis shows the number of miRNAs (m). The y-axis shows α, which is used for penalized regression methods. a. A combination of $(m, \alpha) = (530, 0.4)$ achieved the highest mean accuracy in the test set (blue rectangle). b. A combination of $(m, \alpha) = (470, 0.2)$ achieved the highest mean of mean accuracies among subtypes in the test set (blue rectangle).

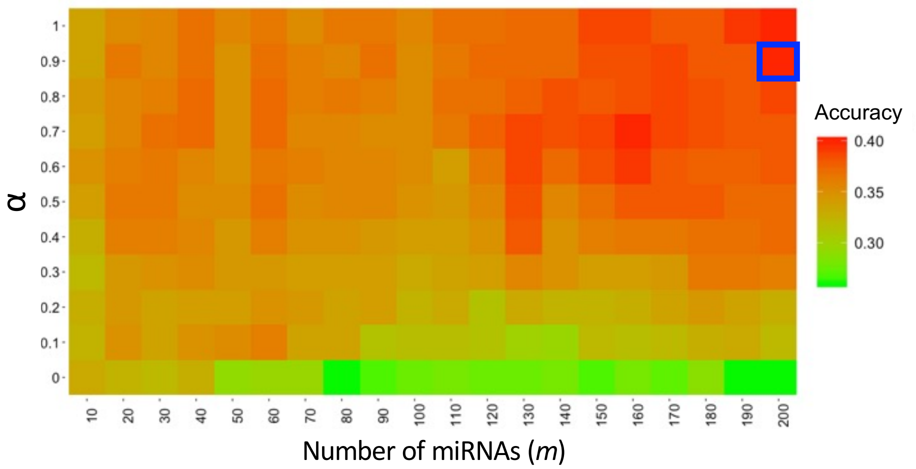

Supplementary Fig. S3. Five-fold cross-validation on a discovery set of Dataset 3.
The x-axis shows the number of miRNAs (m). The y-axis shows α, which is used for penalized regression methods. Combinations of $(m, \alpha) = (200, 0.9)$ and $(200, 1.0)$ achieved the same highest mean accuracy in the test set (blue rectangle).
