# Supplementary material for: Dementia subtype prediction models constructed by penalized regression methods for multiclass classification using serum microRNA expression data
Source: Sci Rep. 2021 Oct 22;11:20947. doi: 10.1038/s41598-021-00424-1 (PMC8536697; doi:10.1038/s41598-021-00424-1)
Supplement: Supplementary file 3 — Supplementary Information. [file 41598_2021_424_MOESM3_ESM.pdf]

Supplementary Information for

Dementia subtype prediction models constructed by penalized regression methods for multiclass classification using serum microRNA expression data

Yuya Asanomi[1], Daichi Shigemizu[1–3*], Shintaro Akiyama[1], Takashi Sakurai[4,5], Kouichi Ozaki[1,3], Takahiro Ochiya[6,7], and Shumpei Niida[1*]

[1] Medical Genome Center, National Center for Geriatrics and Gerontology, Obu, Aichi, Japan

[2] Department of Medical Science Mathematics, Medical Research Institute, Tokyo Medical and Dental University, Tokyo, Japan

[3] RIKEN Center for Integrative Medical Sciences, Yokohama, Japan

[4] Center for Comprehensive Care and Research on Memory Disorders, National Center for Geriatrics and Gerontology, Obu, Aichi, Japan

[5] Department of Cognition and Behavior Science, Nagoya University Graduate School of Medicine, Nagoya, Aichi, Japan

[6] Division of Molecular and Cellular Medicine, Fundamental Innovative Oncology Core Center, National Cancer Center Research Institute, Tokyo, Japan

[7] Department of Molecular and Cellular Medicine, Institute of Medical Science, Tokyo Medical University, Tokyo, Japan

* Correspondence should be addressed to D.S. (daichi@ncgg.go.jp) or S.N. (sniida@ncgg.go.jp)

Construction of prediction models based on different sample sizes of the discovery set

In this study, we constructed four prediction models with different sample sizes of the discovery set, and carefully examined the effect of sample size in the four models. The sample characteristics of each data set are summarized in Supplementary Table S1 (Dataset 1).

We first divided 1594 Japanese individuals into a discovery set of 1061 individuals (672 AD, 59 VaD, 110 DLB, 56 NPH, and 164 CN) and a validation set of 533 individuals (337 AD, 30 VaD, 56 DLB, 28 NPH, and 82 CN) as shown in Supplementary Table S1 (Dataset 1). We examined the accuracy using combinations of m and α, and observed a maximum accuracy of 0.706 at (m, α) = (510, 0.3) in the discovery data set (Supplementary Fig. S1). The final prediction model, Model A, was constructed based on the optimal m and α detected using the entire discovery set. The adjusted model was then evaluated on the validation data set, which achieved an accuracy of 0.707 with 183 miRNAs (Supplementary Table S2). However, this model could not reliably predict non-AD dementia subtypes, and only achieved an average accuracy of each dementia subtype of 0.260, because the model was constructed using a large proportion (63% of total samples) of AD samples (Supplementary Table S3).

To eliminate this bias of AD sample size, we decreased the number of AD individuals used in the discovery data set to a similar sample size as the other dementia subtypes (Supplementary Table S1, Dataset 2) and again examined combinations of m and α through 5-fold cross-validation, as previously described. A maximum accuracy of 0.524 was observed for the combination of (m, α) = (530, 0.4) in the discovery data set (Supplementary Fig. S2a). The final prediction model, Model B, achieved an accuracy of 0.507 in the validation data set when using 140 miRNAs (Supplementary Table S4, called Model B). Although Model B was superior to Model A for each dementia subtype prediction (average accuracy of each dementia subtype, Model A = 0.260, Model B = 0.300), it still showed low predictive abilities for VaD and NPH (Supplementary Table S3).

To further improve the predictive ability in each dementia subtype, we changed the model parameter optimization method from maximizing accuracy in all samples to maximizing mean

accuracy across each dementia subtype. We achieved a maximum mean accuracy with a parameter combination of (m, α) = (470, 0.2) in each dementia subtype (Supplementary Fig. S2b); the final prediction model, Model C, achieved a mean accuracy of 0.507 in the validation set when using 209 miRNAs (Supplementary Table S5). In Model C, the predictive abilities were improved in several dementia subtypes (VaD, DLB, and NPH) compared with Model A and Model B (Supplementary Table S3).

To eliminate the bias of sample size among dementia subtypes, we constructed another a model using the same number of samples in each dementia subtype. The final prediction model, Model D (Supplementary Table S6), achieved an accuracy of 0.398 in the validation data set and a mean accuracy of 0.374 for dementia subtypes (Supplementary Table S3) when using 46 miRNAs. One of the most important strategies in this dementia subtype prediction is to minimize classifying dementia patients as CN (false negative prediction rate for dementia: FN for dementia). Model D achieved a lower FN for dementia than Model B or C and showed a higher predictive ability in each dementia subtype than Model A (Supplementary Table S3).
